# Clinicopathologic Study of Calcifying Fibrous Tumor Emphasizing Different Anatomical Distribution and Favorable Prognosis

**DOI:** 10.1155/2019/5026860

**Published:** 2019-07-02

**Authors:** Jun Zhou, Luting Zhou, Sheng Wu, Ruokun Li, Xiaoqun Yang, Haiming Xu, Saifang Zheng, Anran Wang, Chaofu Wang

**Affiliations:** ^1^Department of Pathology, Ruijin Hospital, Shanghai Jiaotong University School of Medicine, No. 197 Ruijin Er Road, Huangpu District, Shanghai 200025, China; ^2^Department of Pathology, Jinjiang People's Hospital, No. 28 Zhongzhou Road, Jingjiang, Jiangsu 214500, China; ^3^Department of Radiology, Ruijin Hospital, Shanghai Jiaotong University School of Medicine, No. 197 Ruijin Er Road, Huangpu District, Shanghai 200025, China

## Abstract

**Aims:**

Calcifying fibrous tumor (CFT) is a very rare begin fibroblastic tumor featuring a widely anatomical distribution and may mimic various spindle cell tumors. Misdiagnosis and hence mistreatment are likely caused due to unfamiliarity to clinicians or junior pathologists. We collected a relatively large series of CFTs in our institution aiming at further summarizing their clinicopathologic features in Chinese patients and discussing the diagnosis and differential diagnosis in clinical practice.

**Methods:**

Clinicopathologic data of 22 CFTs were retrospectively reviewed. Histologic features were reevaluated and summarized. Immunostaining markers include CD34, SMA, Desmin, keratin, S100, ALK1, CD117, IgG, IgG4, and Ki-67. Follow-up of all cases was performed.

**Results:**

22 CFTs include gastric (n=8), pulmonary (n=2), hepatic (n=2), cervical (n=1), appendiceal (n=1), esophageal (n=1), retroperitoneal (n=1), intra-abdominal (n=1), diaphragmatic (n=1), spermatic cord and scrotum (n=1), anconeal (n=1), mesenteric (n=1), and omental (n=1) lesions. Coexisting hepatocellular carcinoma, pancreatic carcinoma, pheochromocytoma, Castleman disease, and leiomyoma of the uterus and other metabolic or functional disorders were also appreciated. CFT histologically features spindle cells embedded dense hyalinized stroma with scattered psammomatous calcifications and lymphoplasmacytic infiltration and immunohistochemically for CD34. None of any individuals die of CFT per se.

**Conclusion:**

Our study discloses that CFT is a bona fide benign fibroblastic lesion, regardless of its developing location. Involvement of digestive tract seems much more common in the Chinese population. Awareness of the clinicopathologic characteristics of this rare entity and its mimickers contribute to avoiding misdiagnosis and mistreatment in clinical practice.

## 1. Introduction

The calcifying fibrous tumor is an extremely rare benign lesion characteristic of hypocellular bland fibroblastic proliferation embedded in the hyalinized collagenous stroma with chronical inflammation and prominent calcification [1]. This tumor was first reported by Rosenthal and Abdul-Karim as “calcifying fibrous pseudotumor (CFPT) with psammoma bodies” in subcutaneous and deep soft tissues in 2 children [2]. However, increasing cases have been appreciable in various anatomical locations such as the soft tissues, neck, stomach, small intestine, mesentery, peritoneum, mediastinum, adrenal gland, and so on [3–9]. The tumor was primarily regarded as a late stage of inflammatory myofibroblast tumor (IMT) but now has been proposed as a distinct fibroblastic tumor [1, 10, 11]. Individuals may be admitted for an incidentally identified lesion or corresponding clinical syndromes resulting from mass effect. A subset can be multifocal and has some accompanying lesions such as Castleman disease, IMT, vascular anomaly, angiomatoid nodular transformation (SANT) et al. [12–15].

CFT may mimic IMT, fibromatosis, gastrointestinal stromal tumor (GIST), synovial sarcoma, desmoplastic fibroblastoma, fibroma of the tendon sheath, and IgG4-related disease (IgG4 RD) in the given clinical scenario. A misdiagnosis or unnecessary overtreatment is likely rendered due to failing to think of CFT, particularly, arising in some uncommon locations.

Most studies are sporadic case reports and only a few case series systematically depicted clinicopathologic features. However, as far as we know, no study from a single institution has described the features of CFT onset of different locations and frequently concurrent disease. Herein, we collected 23 CFT with distinct anatomical distribution and coexisting disease to further analyze their clinicopathologic features.

## 2. Methods

### 2.1. Patients and Case Selection

This study was approved by the Institutional Review Board at the Department of Pathology, Ruijin Hospital, Shanghai Jiaotong University School of Medicine. A total of 23 CFTs entered our study, including 19 surgical specimens and 4 consultant cases. Each of them has complete clinical data and was reviewed. All hematoxylin and eosin (HE)-stained slides were independently reviewed by 2 experienced pathologists (J. Z. and C.F.W.) and a diagnostic consensus of CFT was reached on every case. Follow-up was performed in the office setting or by telephone interview.

### 2.2. Immunohistochemistry (IHC)

IHC evaluation of consultant case was recorded according to the submitted immunostaining slides. Each surgical specimen was specially resectioned. 4-*μ*m thick sections were taken from 10% formalin-fixed and paraffin-embedded tissue blocks followed by immunohistochemical staining using commercially available antibodies as follows: CD34 (QBEND, prediluted; Dako, Glostrup, Denmark), AE1/AE3 (AE1/AE3, prediluted; Dako, Carpinteria, California, USA), SMA (1A4, 1:200; ZSGB-BIO, Beijing, China), Desmin (D33, prediluted; Dako, Glostrup, Denmark), CD117 (OTIC6, 1:200; ZSGB-BIO, Beijing, China), Dog-1 (SP1, 1:100; LBP, Guangzhou, GD, China), S100 (polyclonal, prediluted; Dako, Glostrup, Denmark), ALK1 (AKL1, prediluted; Glostrup, Denmark), CD138 (EP201, 1:200 dilution; ZSGB-BIO, Beijing, China), CD38 (SPC32, 1:150 dilution; ZSGB-BIO, Beijing, China), IgG (polyclonal, 1:200; ZSGB-BIO, Beijing, China), IgG4 (EP138, 1:200; ZSGB-BIO, Beijing, China), Ki-67 (MIB-1, prediluted; Dako, Glostrup, Denmark), immunoglobulin (Ig) *κ* (polyclonal, 1:1000; Dako, Glostrup, Denmark), and *λ* (polyclonal, 1:1000; Dako, Glostrup, Denmark) light chains. Detection of antibody binding was obtained using the universal immunoperoxidase polymer method (Envision-kit; Dako, Carpinteria, CA, USA). A Dako automated immunohistochemistry system (Dako, Carpinteria, CA, USA) was performed according to the manufacturer's protocol. The IHC results were independently interpreted by 2 experienced pathologists (L. Z. and C.F.W.).

## 3. Results

### 3.1. Clinical Features

**Figure 1:**
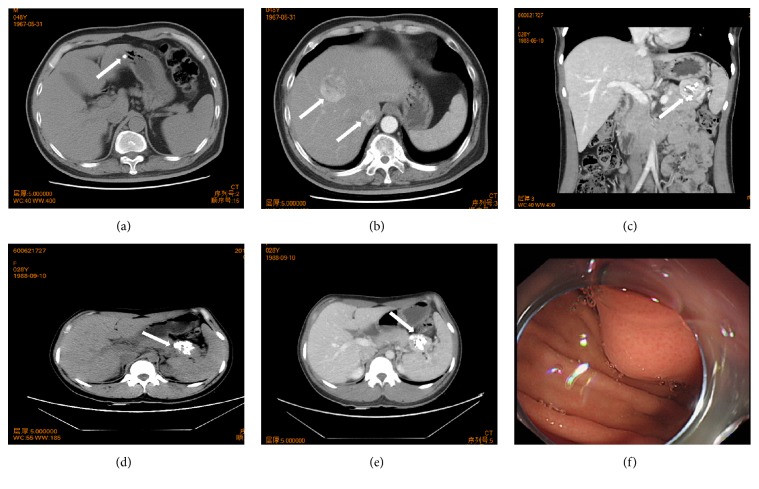
Imaging characteristics of CFT. Axial CT scan showed a soft-tissue density nodule (arrow) with centrally prominent calcification on the gastric wall (case 16) (a); meanwhile, the contrast-enhanced CT indicated two hepatic masses with mild enhancement (arrow; case 16) (b); the coronal CT scan demonstrated a round mass (arrow) with evident calcification in the upper-left abdominal cavity (case 19) (c); axial CT revealed a mass with calcification in the abdominal cavity in proximity to the tail of the pancreas (arrow; case 19) (d); contrast-enhanced CT indicated the well-defined mass mild enhancement peripherally (arrow; case 19) (e); gastroscopy showed a submucosal bulging mass (arrow) with overlying intact smooth mucosa (f).

The main clinical data are summarized in Table 1. Cases contain 13 males and 9 females, ranging in age from 7 to 72 years (mean, 48.5 years; median, 48 years) without a known family history of CFT. The involved location was variable including deep soft tissues in body surface (right neck, n=1; right elbow, n=1), body cavity (mediastinum, n=1; thoracic cavity, n=1; posterior peritoneum, n=1; abdominal cavity, n=1), lung (left upper and lower lung, n=1; left lower lung, n=1), digestive system (stomach, n=8 [Figure 1(a)]; ileum, n=1; colon, n=1; appendix, n=1; liver, n=2), 82% (n=18), and spermatic cord with scrotum (n=1). Most of the cases were solitary other than 4 cases involving colon, stomach, lung, and spermatic cord with scrotum, respectively. CFTs ranged in size from 0.4 cm to 6 cm in greatest dimension (mean, 1.9 cm; median, 1 cm), where the tumors located on the posterior peritoneum or intra-abdominal cavity were slightly larger (4.5 cm, 6 cm in greatest dimension respectively). 68% (n=15) of patients were asymptomatic, most of them found the tumor incidentally when routine physical examination or operation performed for other diseases. A subset of tumors presented with mass effect characterized by pain (cases 5 and 20), abdominal distention (case 1 and 15), hungry feeling (case 8), belching (case 6), and emesis (case 19). Most (86%, n=19) of the objects synchronically had various diseases including cancer, chronic inflammatory or autoimmune disease, functional disorder, or metabolic disease (Table 1); the remaining lesions (18%, n=4) without any observed diseases were located in soft tissues, 75% (n=3) of which were superficial mass. 75% of cases with gastric involvement simultaneously represented chronic superficial gastritis (CSG; cases 6, 7 and 9) or atrophic gastritis (AG; cases 1, 5, and 16). 2 patients with gastric CFT (Figure 1(a)) were also affected by pancreatic carcinoma (PC) and hepatocellular carcinoma (HCC [Figure 1(b)]), respectively. Both 2 cases of CFT involving liver had chronic cholecystitis and cholelithiasis, 1 of which suffered from HCC, chronic hepatitis B, and liver cirrhosis. Castleman disease (CD) was appreciable in the intra-abdominal CFT (Figures 1(c) and 1(d)). A case of the appendiceal lesion had chronic myeloid leukemia. Other coexisting diseases, as depicted in Table 1, include chronic appendicitis, cholelithiasis, diabetes mellitus, chronic cholecystitis, uterine leiomyoma, serous cystadenoma, high blood pressure, herniated intervertebral discs, Hashimoto thyroiditis, pheochromocytoma, pituitary adenoma, Henoch-Schönlein purpura nephritis, ankylosing spondylitis, and polypoid lesion of the gallbladder.

### 3.2. Pathologic Features

**Figure 2:**
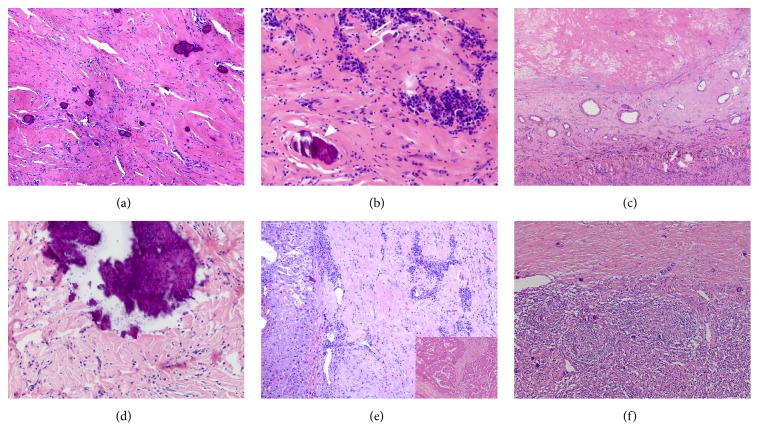
Histologic features of CFT. Spindle cells are embedded in the hyalinized stroma with prominent chronic inflammation and calcification (a; ×100); moderate power shows psammomatous calcification (arrowhead) and abundant plasma cells; Russell's bodies either inside or outside of plasmacytes are frequently appreciable (arrow) (b, ×200); prominent hyalinized stroma results in the paucity of tumor cells and lymphoplasmacytic reaction; note the well-circumscribed border and compressed lung tissues in the lower side of this figure (c; ×50); dystrophic calcification is not uncommon (d; ×200); CFT involves liver; note the well-defined border against the normal hepatic tissue (left side) and the coexisting moderate to low differentiated hepatic carcinoma in other region (inset) (e; ×100); CFT juxtaposes Castleman's disease (lower side) characterized by increased regressed lymphoid follicles and scattered tiny calcifications (f; ×100).

All CFTs were grossly well-circumscribed but unencapsulated; they had a gray-white, firm appearance on a cross-section and were cut with a gritty feeling in varying degree. Morphologically, all of them were characterized by spindle cells embedded in the hyalinized stroma with prominent chronic inflammation and calcification (Figures 2(a) and 2(b)). The tumor cell is wispy with tapering ends in parallel to the collagens; inflammatory cells were mainly composed of plasma cells and few small lymphocytes (Figure 2(b)). Hyaline stroma in 2 cases (cases 18; 20) was dominant resulting in the paucity of tumor cells and lymphoplasmacytic reaction (Figure 2(c)). Sporadic lymphoid follicles in 2 cases (cases 8; 15) appeared as well. Both dystrophic (23%, n=5, Figure 2(d)) and psammomatous (77%, n=17) calcification can be readily found ranging in size from scattered delicate to apparently sheet pattern. Very focally myxoid stroma (case 20), osseous metaplasia (case 15) and entrapped adipose tissue (case 11) were separately found in each of the 3 cases. Mitotic figures were not appreciable in any cases. Morphology of coincident chronic superficial gastritis, atrophic gastritis, chronic appendicitis, chronic cholecystitis, uterine leiomyoma, Hashimoto thyroiditis, and serous cystadenoma correspond to the typical histologic features. The coexisting hepatic carcinoma showed moderate to low differentiated in cases 3 and 16 (Figure 2(e)); pancreatic carcinoma was well-differentiated adenocarcinoma in case 17. Castleman disease was unicentric hyaline vascular variant showing transition with CFT in case 19 (Figure 2(f)).

**Figure 3:**
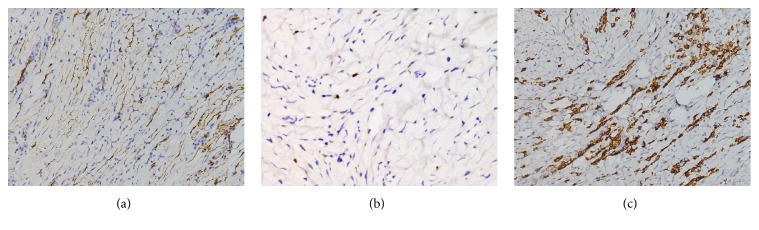
Immunohistochemical features of CFT. The spindle cells express CD34 (a; ×200); proliferative index Ki-67 is typically low (b, ×200); CD38 highlight the abundant plasma cells (c, ×200).

Immunohistochemically, tumor cells of all cases show positive for CD34 (Figure 3(a)), 2 of which were immunoreactive focally. A subset of cases (23%, n=5) displayed focally positive immunostaining for SMA. AE1/AE3, Desmin, CD117, Dog-1, S100, and ALK1 were totally negative. Proliferative index Ki67 were typically as low as 1%, only 2 cases were 2% and 3% each (Figure 3(b)). The plasmacytic component was highlighted by CD38 (Figure 3(c)) and did not show restricted lg*κ* or Ig*λ* expression. Viable positive IgG (range: 5%-80%; mean: 31%, median: 20%) were seen in all cases, but only 7 case (32%) show positive for IgG4 (range: 1%-10%; mean: 3.6%, median: 1.5%).

### 3.3. Treatment and Follow-Up Data

As depicted in Table 1, patients underwent surgical resection alone or along with otherwise operation. The endoscopic submucosal excavation was performed in the cases with gastric involvement when they were incidentally found by physical examination. 3 patients (cases 3, 11, and 16) received chemotherapy against their corresponding carcinomas. All follow-up data (range, 9 to 63 months; mean, 24 months; median, 25 months) were available until this article was written. 2 patients (case 3 and 17) succumbed to hepatic and pancreatic carcinoma respectively but none directly died of CFT.

## 4. Discussion

CFT is a very rare benign tumor that can seemingly develop in a variety of anatomic location. The deep soft tissue of superficial body was supposed to be the most involved location, such as extremities, trunk, inguinal and scrotal areas, or head and neck [16]. However, Chorti et al. reviewed 157 CFTs reported in international literature disclosing that the most common involved locations are stomach (18%) and small intestine (8.7%) followed by pleura (9.9%), neck (6.2%), mesentery (5%), mediastinum (5%), and peritoneum (6.8%) [17]. Our series also shows that lesions developing in the gastrointestinal tract account 50% (11/22) of the total, of which the gastric tumor is in the majority (36%, 8/22), followed by the small intestine (4.5%, n=1/22) and appendix (4.5%, n=1/22). That appears to be plausible because the frequency of CFTs founded, according to our experience, in the Chinese population tend to be way more predominant compared to the deep soft-tissues lesions. The proportion of lesions arising in the mediastinum (4.5%, n=1/22) is similar to the reported statistics, but pleura (4.5%, n=1/22) and peritoneum (4.5%, n=1/22) a little bit lower in our research group. Only 9.1% of patients have soft tissues of the superficial body involved, which is significantly fewer than what one traditionally considered in the western. Both hepatic and pulmonary CFT are exquisitely rare, and no more than 3 cases were reported each [17–20]. In addition, 2 pulmonary CFTs with multiple involvements in our series are very unique and 1 hepatic CFT coincident with HCC is not yet found in the literature.

The symptom of CFTs is variable due to its heterogeneous location distribution. 30.57% of cases demonstrate various symptoms characterized by nonspecific, locally or systemically [17]. As to our cases, most of them were found incidentally and few cases present local symptom due to their mass effects. Scattered literature has reported a subset of CFTs accompanies some distinct entities, as mentioned above. Kuo et al. described 5 out of 10 SANTs with abdominal disseminated calcifying fibrous tumors sharing features overlapping with IgG4 RD [21]. Scattered cases also demonstrate higher IgG4: IgG ratio in tissues and elevated serous IgG, IgG4 ratio, suggesting their possible association with IgG4 RD [22–24]. However, other reports did not show any increased positivity IgG4 or IgG: IgG4 and failed to provide straightforward evidence of the relationship between CFT and IgG4 RD [25, 26]. Additionally, inflammatory infiltrate in CFTs is generally lesser than that in IgG4 RD and the representative obliterative phlebitis of IgG4 RD is almost invisible in CFT [26]. In our series, although some cases imparted proportion of positive IgG in a varying degree ranging from 5%-80%, the number of IgG4 immunostaining was not virtually significant each case; that is far from the diagnostic criterion of IgG4 RD [27]. Accordingly, not every CFT, even if a subset, is directly connected with IgG4 RD. There are sometimes histologic overlaps between CFT and IMT and hence it was proposed by some researchers that CFT represents a late sclerosing stage of IMT [10]. However, IMT is typically more cellular, less collagenous and lacks calcification as well as characteristically ALK expression due to its translocation. Therefore, certain distinct histologic immunohistochemical, genetical, and electron microscopic features investigated by other researchers did support the proposal considering them as the same entity [28, 29]. None of our CFTs had history suffering from IMT and hared evident morphologic and immunohistochemical overlap between them, likewise, suggesting their different nature.

We found quite a few CFTs, particularly the one arising in body cavity or viscera, have coexisting various diseases in our research group that is not be noticed yet. The reason for this scenario ignored is likely due to the sporadic report in the literature. These diseases are generally characterized by chronic local inflammations, consumption, or metabolic disorders in our series. Given that the chronic inflammatory infiltrates and dystrophic calcification is the typical pathologic features, it is plausible that CFTs may associate with the locally dysregulated nutritional status. However, the real links between them are still obscure and need more series of cases to be analyzed. Virtually, the neoplastic nature of CFT remains controversial, although has been described as a begin tumor in WHO classification of tumors of soft tissue and bone [1]. Mehrad et al. investigated the gene status in 3 cases of CFT of the pleura (CFTP) using whole-exome sequencing and identified unique and novel deleterious mutations involving* ZN717*,* FRG1*, and* CDC27* as well as a large loss on chromosome 6 and significant copy number losses on chromosome, all of which were supposed to contribute to CFTP tumorigenesis. However, more molecular studies need to be performed to confirm the real drive mutations or other key genetic alterations.

In addition to IgG4 RD and IMT described above, the differential diagnosis includes fibromatosis, synovial sarcoma, desmoplastic fibroblastoma, fibroma of the tendon sheath, and GIST. Some fibromatosis may present prominent hyaline stroma instead of a typical cellular fascicular configuration, mimicking CFT; a diagnosis of fibromatosis usually means the intermediate biologic behavior characterized by infiltration of adjacent normal tissues resulting to arduously complete excision and frequently local recurrence [30]. But fibromatosis usually has an ill-defined border and lacks distinct lymphoplasmic infiltration and calcification as well as nuclear *β*-catenin positivity [31, 32]. A subset of synovial sarcoma can be calcified like CFT clinically; a diagnosis of synovial sarcoma means the high risks of local recurrence and metastasis and frequent adjuvant therapy such as irradiation or chemotherapy [33]. But histologically highly cellularity, viable expression of keratin and distinctive* SYT *translocation can easily tell them apart [34]. Both desmoplastic fibroblastoma and fibroma of tendon sheath feature well-demarcated, multinodular architecture and hypocellular spindle cells embedded in the hyalinized stroma; but the distinct developing location and deficiency of prominent inflammation or calcification can distinguish them. GISTs should routinely enter the differential diagnosis with visceral CFTs for its various morphologic pattern. Although GISTs often express CD34 as well, CFTs are consistently negative for CD117 and DOG-1, the characteristic markers of GIST.

In conclusion, CFT is an exquisitely rare benign tumor with a favorable prognosis, regardless of its anatomic location. We have reported one of the largest series of CFT focusing its distinct clinicopathologic features. Our study further shows its wide location distribution and predilection to develop in the digestive tract in the Chinese population. Various coexisting diseases are frequently appreciated, particularly the visceral CFTs. To be acquainted with CFT's clinicopathologic features contributes to avoiding misdiagnosis and unnecessary treatment.

## Figures and Tables

**Table 1:** Clinicopathologic features and follow-up of calcifying fibrous tumors.

case	Gender/Age (years)	Location	Size (cm×cm×cm)	Presentation	Coexisting disease	Treatment	Follow-up
1	M/65	Stomach, subserosa	3×3×1	abdominal distension for 30 yr. with aggravation for 0.5 yr.	AG	CR	AWOD, 35 mon.
2	F/59	Appendix, muscularis	1×0.5×0.5	incidental	CA; CL; DM; CML	CR	AWOD, 34 mon.
3	M/56	Liver	2×1.5×1	Incidental	HCC; CHB; LC; CC	CR+chemotherapy	DOCD, 12 mon.
4	F/52	Colon, mesentery and serosa	0.6×0.5×0.3; 0.5×0.5×0.3	Incidental	ULM; SCA	CR	AWOD, 25 mon.
5	M/58	Stomach, submucosa	2.5×2×1.6	Pain in upper abdomen for 4 mon.	AG,CL,	CR	AWOD, 23 mon.
6	M/44	Stomach, submucosa	0.4×0.3×0.2 0.8×0.6×0.5	Belching for 7 yr. with aggravation for 3 yr.	CSG, DM, HT	CR	AWOD, 20 mon.
7	M/50	Stomach, submucosa	0.5×0.3×0.3	Incidental	CSG, HBP, HIVD	CR	AWOD, 19 mon.
8	F/42	Stomach, submucosa	1.1×0.8×0.5	Frequent hungry feeling	AG	CR	AWOD, 15 mon.
9	M/45	Stomach, submucosa	0.7×0.5×0.5	Incidental	CSG	CR	AWOD, 13 mon.
10	F/46	thoracic cavity, parietal pleura	3×2×1.3	Incidental	None	CR	AWOD, 11 mon.
11	F/23	Ileum, mesentery	2×1.5×1	Incidental	PCC; PA	CR	AWOD, 10 mon.
12	M/31	Mediastinum	4×3×2	Incidental	None	CR	AWOD, 9 mon.
13	F/65	Left upper and lower lung	2×1×1 1×0.8×0.5 0.6×0.5×0.5	Incidental	HSPN	CR	AWOD, 37 mon.
14	M/7	Right Neck	4×3×1.5	Painless mass on the right neck	None	CR	AWOD, 28 mon.
15	M/43	Posterior peritoneum	4.5×3.5×3	epigastric distress	CC; HBP	CR	AWOD, 63 mon.
16	M/49	Stomach, muscularis	0.7×0.5×0.5	Incidental	HCC; CC; AS; DM; HBP	CR+chemotherapy	AWOD, 41 mon.
17	M/56	Stomach, muscularis	1×0.5×0.5	incidental	PC	CR+chemotherapy	DOCD, 38 mon.
18	M/46	Left lower lung	3.5×2.7×2.2	Incidental	HBP	CR	AWOD, 33 mon.
19	F/29	Abdominal cavity	6×5×3	Recurrent emesis and abdominal pain for 9 d.	CD	CR	AWOD, 28 mon.
20	F/58	Right elbow	3.4×1×0.6	Mass for 0.5 yr. and pain for 1 mon.	None	CR	AWOD, 25 mon.
21	F/72	liver	2×1×1	Incidental	CC, CL	CR	AWOD, 12 mon.
22	M/70	Left spermatic cord; left scrotum	2×1.5×1 1×1×0.5 0.6×0.5×0.5	incidental	VC	CR	AWOD, 9 mon.

AWOD, alive without disease; DOCD, die of coexisting disease; CR, complete resection; ER, endoscopic resection; AG, atrophic gastritis; CA, chronic appendicitis; CL, cholelithiasis; DM, diabetes mellitus; CML, chronic myeloid leukemia; HCC, hepatocellular carcinoma; CHB, chronic hepatitis B; LC, liver cirrhosis; CC, chronic cholecystitis; ULM, uterine leiomyoma; SCA, serous cystadenoma; CSG, chronic superficial gastritis, HBP, high blood pressure; HIVD, herniated intervertebral discs; HT, Hashimoto thyroiditis; PCC, pheochromocytoma; PA, pituitary adenoma; HSPN, Henoch- *Schönlein* purpura nephritis; AS, ankylosing spondylitis; PLG, polypoid lesion of gallbladder; PC, pancreatic cancer; CD, Castleman disease; VC, varicocele.

## Data Availability

The data is available through contact by the corresponding author Prof. Chaofu Wang if there are no commercial interests.
